# Combined effects of vitamin D and cumulative dietary risk score on fatty liver and mortality in vulnerable individuals: a prospective analysis from the UK Biobank

**DOI:** 10.1007/s11357-025-01762-y

**Published:** 2025-07-01

**Authors:** Xiaoyan Wang, Yongqi Liang, Chenxi Jin, Jingjing Liang, Yining Xu, Xianbo Wu, Mengchen Zou

**Affiliations:** 1 Department of Endocrinology and Metabolism, Nanfang Hospital, Southern Medical University, 1838 Guangzhou Road North, Guangzhou, 510515 China; 2 Department of Occupational Health and Medicine, School of Public Health, Southern Medical University, Guangzhou, China

**Keywords:** All-cause mortality, Cumulative dietary risk score, Frailty phenotype, Metabolically associated fatty liver disease, Vitamin D

## Abstract

Frailty is a global public health issue associated with multiple adverse outcomes in later years, however, studies have shown exercise, diet, and vitamin D supplements can help reverse frailty. There is a lack of further risk stratification and evidence for secondary interventions in populations that have undergone primary prevention. This was a prospective cohort study involving 53106 physically active vulnerable individuals from the UK Biobank. We combined vitamin D with cumulative dietary risk scores to evaluate its predictive value. Correlation and predictive power were assessed using restricted cubic spline (RCS) analysis, corrected Cox models, stratified analysis, Kaplan–Meier analysis, and Harrell's c-index. During 12.7 years of follow-up, there were 870, 1317 new-onset metabolic dysfunction-associated fatty liver disease (MAFLD) and mortality. RCS analysis revealed a J-shaped negative or positive correlation in vitamin D and dietary risk score respectively. The hazard ratios (95% confidence interval) for MAFLD and mortality in the vitamin D deficiency and high dietary risk groups were 1.80 (1.45–2.24) and 1.99 (1.67–2.38), compared to those with no vitamin D deficiency and low dietary risk. Kaplan–Meier analysis suggested vulnerable individuals with vitamin D deficiency and a poor diet were at a higher risk of disease in the future. Further, separate analyses of vitamin D and dietary risk scores did not demonstrate better efficacy. The combination of vitamin D and dietary risk score offers a more effective method for assessing disease risk in vulnerable populations. This provides evidence for secondary guidance following exercise interventions, which is significant for older population.

**Graphical abstract:**

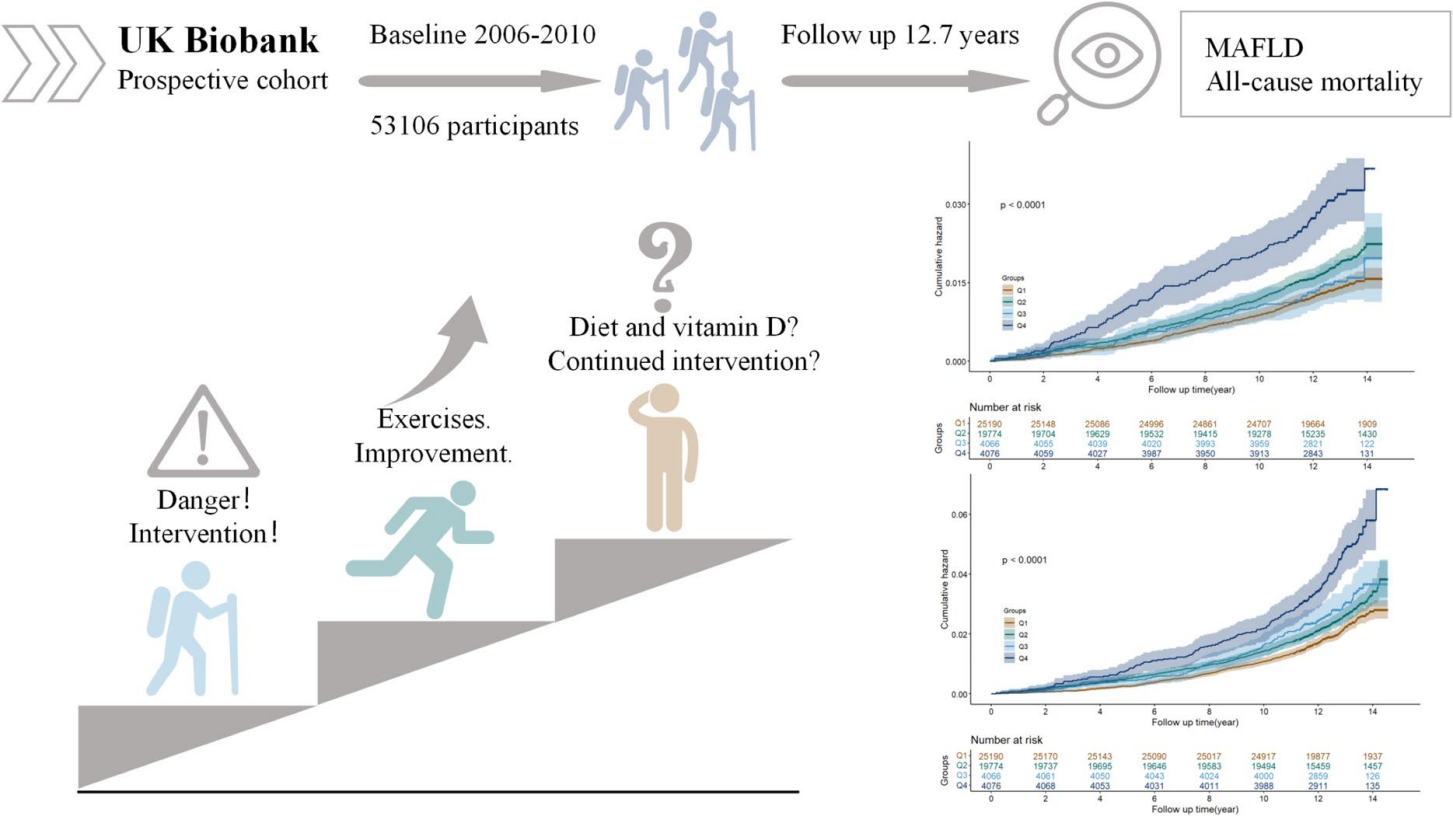

**Supplementary Information:**

The online version contains supplementary material available at 10.1007/s11357-025-01762-y.

## Introduction

Frailty is a clinically identifiable condition characterized by reduced physiological reserve and increased vulnerability to a variety of adverse health outcomes [1]. As populations age, frailty is becoming more prevalent and poses a significant burden on public health. According to the modified Fried frailty phenotype, 9.9% of individuals over 50 years old are frail, while 44.2% are in the early stages of frailty, with these proportions varying across different regions and age groups [2]. Frail individuals are more likely to experience falls, disabilities, hospitalizations, long-term care needs, metabolic disorders, multimorbidity, and higher mortality rates [3]. Frailty is a dynamic, multi-dimensional condition, and numerous studies have shown that interventions such as exercise, diet, and vitamin D supplements—either alone or in combination—may help reverse frailty [4, 5], although some studies have reported inconsistent findings due to the contents and duration of the intervention [6, 7]. However, the majority of research supports exercise as a recommended approach, as it effectively improves bone density, muscle strength, metabolism, and immunity, leading to better physical health and improved disease outcomes [8].

Vitamin D is primarily produced in the skin (80%−90%) after exposure to sunlight, with a smaller amount (10%−20%) obtained through dietary sources or supplements [9]. Older adults are often vitamin D deficient due to reduced sun exposure, decreased physical activity, insufficient dietary intake, and diminished kidney function [9]. While vitamin D was once thought to primarily affect the musculoskeletal system, studies have shown that it also plays a role in the kidneys, liver, and immune system [10]. For instance, vitamin D has been found to enhance liver function by stimulating vitamin D receptors in the liver [11]. Further, metabolic fatty liver is closely linked to conditions such as diabetes, cirrhosis, liver failure, and increased mortality [12], so trace elements can also influence the overall health of the body. The recommended and minimum vitamin D intake levels vary across regions and populations, and precise assessments in vulnerable groups remain unclear [13]. According to the Global Burden of Disease Study, a significant proportion of global mortality can be attributed to poor dietary habits, including inadequate intake of whole grains, oily fish, nuts, fruits, and vegetables, as well as excessive consumption of salt, red meat, processed meats, and sugary drinks [14]. Cumulative dietary risk scores effectively encompass these items and are not strictly confined to specific diseases or regions. Due to the variety of methods used to assess diet quality, drawing consistent conclusions is challenging [15]. The effectiveness of dietary risk scores in assessing frailty remains uncertain. Moreover, previous assessments of diet quality have often been based on categorized data rather than continuous measures, which has limited our understanding of dietary patterns, including whether there is a linear relationship between diet and health outcomes [16], and this will be further explored in our research.

In this study, we combined the analysis of vitamin D levels with a continuous dietary risk score to assess health outcomes in frail individuals. Whether vitamin D and dietary habits pose significant health risks in vulnerable people undergoing primary prevention has been insufficiently studied. Furthermore, there is a lack of research on the combined analysis of multiple indicators.

## Methods

### Study design and participants

This is a prospective, population-based cohort study involving over half a million participants who were recruited and enrolled in the UK Biobank (UKB) between 2006 and 2010. Each participant completed a touchscreen questionnaire, underwent a nurse-led interview, and had physical measurements taken. All participants provided written informed consent for data collection, analysis, and linkage [17]. The study received ethical approval and is part of the UK Biobank project (NHS National Research Ethics Service 16/NW/0274) [17].

To simplify, we firstly excluded participants who were not in a state of pre-frailty or frailty. The frailty phenotype was defined using the modified Fried criteria, which includes five components: unintentional weight loss, fatigue, low grip strength, low physical activity, and slow walking speed (Table S1). Participants not engage in regular physical activity were also excluded (regular physical activity was defined as at least 150 min/week of moderate activity [50%−70% of the maximum heart rate after exercise], or 75 min/week of vigorous activity [70%−85% of the maximum heart rate after exercise], or an equivalent combination. The maximum heart rate is calculated using the formula: 220 minus your age [in years]) [18]. Additionally, individuals diagnosed with metabolically associated fatty liver disease (MAFLD) prior to recruitment, as well as those with missing vitamin D, were excluded from the study. We further excluded participants with a history of cancer and those under the age of 20, as well as those with unknown information regarding ethnicity, smoking, alcohol consumption, and sleep habits. Ultimately, 53,106 participants were included in the analysis. Detailed information regarding the participants excluded is provided in **Fig. S1**.

### Outcomes

Study outcomes were ascertained through the linkage of National Health Service records, including hospital inpatient data and death register records. The details of the linkage procedure can be found at https://www.ukbiobank.ac.uk/.

The primary outcome of this study was MAFLD. By using the International Classification of Diseases, 10th revision (ICD-10) and the Expert Panel Consensus Statement, MAFLD was defined as ICD-10 code K76.0 (fatty [change of] liver, not elsewhere classified) and K75.8 (other specified inflammatory liver diseases).

The secondary outcome was all-cause mortality, and death outcomes were identified through linkage to national death registries. Detailed information can be found at link https://biobank.ndph.ox.ac.uk/~bbdatan/DeathSummaryReport.html.

### Vitamin D, cumulative dietary risk score and other covariates

Vitamin D was analyzed using a CLIA analysis on a DiaSorin Ltd and units of measurement are nmol/L, which was taken on an empty stomach. The cumulative dietary risk factor score was derived from nine food items previously included in the UK and European dietary guidelines: processed meat, red meat, total fish, milk, spread type, cereal intake, salt added to food, water, and fruits and vegetables [19]. As food item data were collected using varying frequencies of consumption (e.g., categorical responses such as “never”, “less than once a week”, “once a week”, or “almost every day”), each food item was dichotomized into meeting or not meeting the recommendations. These cutoffs were derived from the UK and European food-based dietary guidelines, including the Eatwell Guide and the Food-Based Dietary Guidelines from the European Food Safety Authority, or based on median intake for food items without specific recommendations [16]. The cumulative dietary risk factor score is a continuous variable, with a score ranging from 0 (most healthy) to 9 (least healthy). More details are available in Table S2.

In addition, socio-demographic variables included age, sex, and race (categorized as white or non-white). Lifestyle habits encompassed smoking, alcohol consumption, sleep quality, and body mass index (BMI). Smoking status and alcohol consumption was classified as never, ever, and currently. Sleep quality was categorized as rarely experiencing insomnia, sometimes experiencing insomnia, and often experiencing insomnia. BMI was classified as underweight, normal weight, or overweight, with cut-off points at 19.5 kg/m^2^ and 25 kg/m^2^. Disease-related measures included uncontrolled hypertension (defined as systolic blood pressure ≥ 140 mmHg or diastolic blood pressure ≥ 90 mmHg) and diabetes (defined as fasting blood glucose ≥ 7.0 mmol/L or glycated hemoglobin ≥ 6.5%). Other metabolic indicators included alanine aminotransferase (ALT), aspartate aminotransferase (AST), total bilirubin, creatinine, urea, and C-reactive protein. The quality control procedure for blood samples can be found at https://biobank.ndph.ox.ac.uk/showcase/showcase/docs/biomarker_issues.pdf.

### Statistical analysis

Firstly, we divided the data into two parts based on vitamin D deficiency, using a cutoff point of 30 nmol/L. Using the median cumulative dietary risk as a reference, we further divided the data into two parts with a cutoff point of 4. This resulted in four groups (Q1-Q4): 1) no vitamin D deficiency and lower cumulative dietary risk score, 2) no vitamin D deficiency and higher cumulative dietary risk score, 3) vitamin D deficiency and lower cumulative dietary risk score, and 4) vitamin D deficiency and higher cumulative dietary risk score. Baseline information for participants was then collected according to these preliminary subgroups, including social demographic characteristics, living habits, health status, biochemical indicators. Mean interpolation was used in missing continuous variables, and missing categorical variables were excluded. Continuous variables were expressed as mean and standard deviation (SD), and categorical variables were expressed by frequency and proportion. The t test (continuous variable) or chi-square test (categorical variable) were used to assess the differences between subjects. And the dose–response relationships between vitamin D levels, cumulative dietary risk scores, and the risk of incident MAFLD and mortality were assessed using restricted cubic splines (RCS) with four knots in the adjusted model.

Next, we constructed a Cox proportional hazards model to explore the effects of vitamin D levels and cumulative dietary risk scores on MAFLD and mortality in pre-frail and frail individuals who had undergone exercise interventions. Using the first interval (Q1) as the reference group, we calculated hazard ratio (HR), 95% confidence intervals (CI), and p-values. Model 1 was unadjusted; Model 2 was adjusted for age and sex; Model 3 was adjusted for all variables, including age, sex, race, smoking, alcohol consumption, sleep, BMI, hypertension, diabetes, and biochemical indicators (ALT, AST,total bilirubin, creatinine, urea, and C-reactive protein). Additionally, we divided vitamin D levels and cumulative dietary risk scores into four intervals respectively to further explore the impact of individual measures on health outcomes, and the trend test was performed using the interval median value. Outcomes were analyzed both categorically (different interval) and continuously (with vitamin D measured per 1-unit increment and cumulative dietary risk scores per 1-unit increment). In order to evaluate the predictive power of each indicator, we calculate the Harrell's c index over time. In addition, we performed a subgroup analysis based on age and sex to further compare the C-index.

Further, the interaction between vitamin D levels or cumulative dietary risk scores, with factors such as age, sex, ethnicity, smoking, alcohol consumption, sleep, and BMI on MAFLD and all-cause mortality was analyzed using additive product terms in the Cox model. We further conducted subgroup analysis by age (< 60 years vs. −  > 60 years), sex (male vs. female), ethnicity (White vs. Non-White), smoking status (never, ever, and currently), alcohol consumption (never, ever, and currently), insomnia (rarely, sometimes, and often), BMI (< 25 kg/m2 vs. >  − 25 kg/m2) [19]. In addition, the log-rank test was performed to compare the cumulative incidence of study outcomes across the four groups and survival curves were developed. We also conducted sensitivity analyses. First, we excluded participants who experienced an outcome event within the first two years of follow-up to rule out potential reverse causality. Second, we excluded participants with very low (< 1%) or very high (> 99%) vitamin D levels to eliminate the influence of extreme values on the average. Additionally, we excluded participants in the acute phase of infection to minimize the potential bias from frailty. Finally, we used median interpolation instead of mean interpolation.

All tests were two-tailed, and p < 0.05 was considered significant. Statistical analyses were conducted using R studio software, version 4.3.2 (Core Team. 2023, Vienna, Austria).

## Results

### Baseline characteristics of participants

According to the modified frailty phenotype proposed by Fried, a total of 51523 participants were classified as pre-frail, while 1583 participants were classified as frail. During the median follow-up of 12.67 years, 870 (1.64%) of participants developed MAFLD, and 1317 (2.48%) died. Based on the combined grouping of vitamin D and cumulative risk scores, the frailty status of participants in each group, along with the statistics for MAFLD and death events during the follow-up period, are presented in Table S3. Overall, according to baseline characteristics, participants without vitamin D deficiency and lower cumulative dietary risk scores were more likely to be white women, who had better smoking and drinking habits, lower ALT and C-reactive protein levels, and demonstrated better weight, blood pressure, and blood glucose management (Table 1).

**Table 1:** Baseline characteristics of included participants in the UK Biobank

Characteristics	All	Q1	Q2	Q3	Q4	p.overall
	N = 53106	N = 25190	N = 19774	N = 4066	N = 4076	
Age,years	54.5 (8.22)	55.3 (8.19)	54.1 (8.18)	53.8 (8.22)	52.5 (7.96)	< 0.001
Sex,n(%)						< 0.001
Female	29057 (54.7%)	15780 (62.6%)	8946 (45.2%)	2503 (61.6%)	1828 (44.8%)	
Male	24049 (45.3%)	9410 (37.4%)	10828 (54.8%)	1563 (38.4%)	2248 (55.2%)	
Ethnic_group,n(%)						< 0.001
White	47577 (89.6%)	23058 (91.5%)	17944 (90.7%)	3305 (81.3%)	3270 (80.2%)	
Non-white	5529 (10.4%)	2132 (8.46%)	1830 (9.25%)	761 (18.7%)	806 (19.8%)	
Drinking,n(%)						< 0.001
Never	1530 (2.88%)	731 (2.90%)	390 (1.97%)	230 (5.66%)	179 (4.39%)	
Former	1437 (2.71%)	715 (2.84%)	422 (2.13%)	169 (4.16%)	131 (3.21%)	
Current	50139 (94.4%)	23744 (94.3%)	18962 (95.9%)	3667 (90.2%)	3766 (92.4%)	
Smoking,n(%)						< 0.001
Never	48774 (91.8%)	23883 (94.8%)	17758 (89.8%)	3750 (92.2%)	3383 (83.0%)	
Former	2849 (5.36%)	747 (2.97%)	1365 (6.90%)	204 (5.02%)	533 (13.1%)	
Current	1483 (2.79%)	560 (2.22%)	651 (3.29%)	112 (2.75%)	160 (3.93%)	
Insomnia,n(%)						0.165
Occasional	14160 (26.7%)	6686 (26.5%)	5209 (26.3%)	1151 (28.3%)	1114 (27.3%)	
Sometimes	25565 (48.1%)	12120 (48.1%)	9548 (48.3%)	1931 (47.5%)	1966 (48.2%)	
Often	13,381 (25.2%)	6384 (25.3%)	5017 (25.4%)	984 (24.2%)	996 (24.4%)	
BMI,kg/m^2^,n(%)						< 0.001
underweight	257 (0.48%)	125 (0.50%)	81 (0.41%)	25 (0.61%)	26 (0.64%)	
normalweight	20286 (38.2%)	10604 (42.1%)	7007 (35.4%)	1400 (34.4%)	1275 (31.3%)	
excessweight	32563 (61.3%)	14461 (57.4%)	12686 (64.2%)	2641 (65.0%)	2775 (68.1%)	
Hypertension,n(%)						0.041
No	30537 (57.5%)	14622 (58.0%)	11330 (57.3%)	2298 (56.5%)	2287 (56.1%)	
Yes	22569 (42.5%)	10568 (42.0%)	8444 (42.7%)	1768 (43.5%)	1789 (43.9%)	
Diabetes,n(%)						< 0.001
No	51310 (96.6%)	24481 (97.2%)	19086 (96.5%)	3853 (94.8%)	3890 (95.4%)	
Yes	1796 (3.38%)	709 (2.81%)	688 (3.48%)	213 (5.24%)	186 (4.56%)	
ALT,U/L	22.6 (13.1)	21.6 (12.0)	23.1 (13.1)	23.0 (13.8)	25.5 (17.7)	< 0.001
AST,U/L	25.9 (9.17)	25.8 (8.46)	26.0 (9.04)	25.7 (10.1)	26.8 (12.5)	< 0.001
TBil,umol/L	9.39 (4.54)	9.42 (4.61)	9.51 (4.49)	8.97 (4.48)	9.13 (4.34)	< 0.001
Urea,mmol/L	5.34 (1.27)	5.34 (1.24)	5.44 (1.29)	5.06 (1.21)	5.13 (1.32)	< 0.001
Creatinine,umol/L	71.9 (14.8)	70.3 (14.0)	74.3 (15.3)	68.5 (13.2)	72.8 (16.3)	< 0.001
CRP,mg/L	2.07 (3.58)	1.94 (3.59)	2.14 (3.54)	2.16 (3.67)	2.36 (3.57)	< 0.001

*ALT* alanine aminotransferase, *AST* aspartate aminotransferase, *TBiL* total bilirubin, *Urea* urea, *Creatinine* creatinine, *CRP* highly sensitive C-reactive protein, Continuous variables are presented as the mean and SD, category variables are described as the frequency and percentage

### RCS analysis

**Figure. 1:**
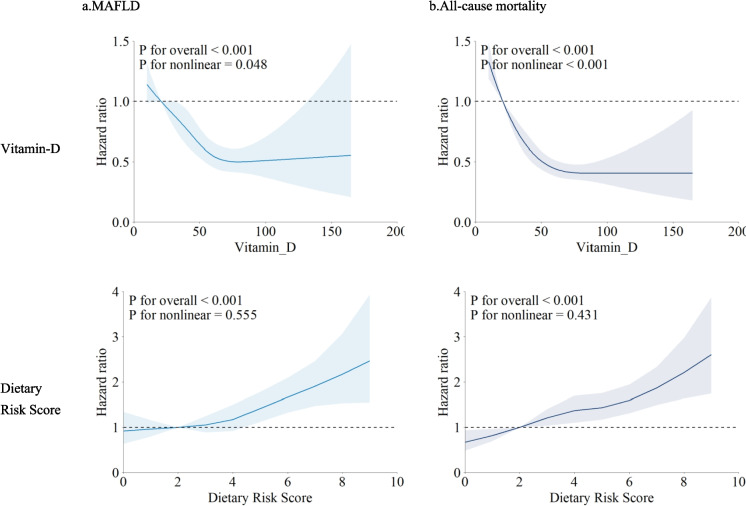
Non‑linear relationships of Vitamin-D and Dietary Risk Score for MAFLD (**a**) and all-cause mortality (**b**). Hazard ratios were adjusted for age and sex

A J-shaped association (p for non-linear < 0.05) was observed between vitamin D levels and the risk of MAFLD and mortality events, with the risk of outcomes increasing across the vitamin D range. Similarly, a monotonically decreasing J-shaped association (p for overall < 0.001) was found between the cumulative dietary risk score and the risk of MAFLD and mortality events (Fig. 1).

### Relationship between vitamin D and cumulative dietary risk score with MAFLD, and all-cause mortality

Table 2 presents the association between vitamin D levels, cumulative dietary risk scores, and the risk of MAFLD and all-cause mortality. After fully adjusting for sociodemographic factors (age, sex, ethnicity), lifestyle factors (alcohol and smoking status, sleep status, BMI), health status (hypertension, diabetes), and metabolic markers (ALT, AST, total bilirubin, urea, creatinine, C-reactive protein), we found that individuals with prefrailty or frailty who were deficient in vitamin D and had higher cumulative dietary risk scores had a significantly increased risk. Specifically, compared to the reference group (Q1) in the fully adjusted model, the HR values and 95% confidence intervals of Q2-4 for MAFLD were 1.22(1.05–1.42), 0.97(0.74–1.29), 1.80(1.45–2.24) respectively. The HR values and 95% confidence intervals for all-cause mortality were 1.07(0.94–1.21), 1.51(1.24–1.85), 1.99(1.67–2.38).

**Table 2:** Association of their combination, individual Vitamin-D or Dietary Risk Score with MAFLD and all-cause mortality

Characteristic	Crude Model (Model 1)	Partially Adjusted Model (Model 2)	Fully Adjusted Model (Model 3)
	HR (95% CI)*p* value	HR (95% CI)*p* value	HR (95% CI)*p* value
MAFLD			
Combination			
Quartile 1	1.00(Ref)	1.00(Ref)	1.00(Ref)
Quartile 2	1.33(1.14–1.54)***	1.32(1.14–1.54)***	1.22(1.05–1.42)**
Quartile 3	1.09(0.83–1.44)	1.12(0.85–1.48)	0.97(0.74–1.29)
Quartile 4	2.24(1.82–2.76)***	2.21(1.87–2.85)***	1.80(1.45–2.24)***
Vitamin-D			
Quartile 4	1.00(Ref)	1.00(Ref)	1.00(Ref)
Quartile 3	1.23(0.99–1.52)	1.23(0.99–1.52)	1.13(0.92–1.40)
Quartile 2	1.50(1.23–1.83)***	1.51(1.24–1.85)***	1.28(1.04–1.57)*
Quartile 1	1.80(1.48–2.19)***	1.85(1.52–2.25)***	1.47(1.20–1.79)***
*p* for trend	< 0.001	< 0.001	< 0.001
Dietary Risk Score			
Quartile 1	1.00(Ref)	1.00(Ref)	1.00(Ref)
Quartile 2	1.04(0.86–1.27)	1.03(0.85–1.26)	0.97(0.80–1.18)
Quartile 3	1.36(1.12–1.65)**	1.35(1.11–1.64)**	1.22(1.01–1.48)*
Quartile 4	1.65(1.37–1.99)***	1.64(1.35–1.99)***	1.38(1.14–1.68)**
*p* for trend	< 0.001	< 0.001	< 0.001
all-cause mortality			
Combination			
Quartile 1	1.00(Ref)	1.00(Ref)	1.00(Ref)
Quartile 2	1.21(1.07–1.37)**	1.18(1.04–1.34)**	1.07(0.94–1.21)
Quartile 3	1.41(1.15–1.72)***	1.65(1.35–2.01)***	1.51(1.24–1.85)***
Quartile 4	2.11(1.78–2.50)***	2.49(2.10–2.96)***	1.99(1.67–2.38)***
Vitamin-D			
Quartile 4	1.00(Ref)	1.00(Ref)	1.00(Ref)
Quartile 3	1.18(0.99–1.40)	1.17(0.98–1.39)	1.17(0.99–1.38)
Quartile 2	1.40(1.19–1.65)***	1.49(1.27–1.76)***	1.45(1.23–1.71)***
Quartile 1	1.75(1.49–2.04)***	2.07(1.77–2.42)***	1.87(1.60–2.20)***
*p* for trend	< 0.001	< 0.001	< 0.001
Dietary Risk Score			
Quartile 1	1.00(Ref)	1.00(Ref)	1.00(Ref)
Quartile 2	1.14(0.98–1.33)	1.08(0.92–1.26)	1.02(0.87–1.19)
Quartile 3	1.28(1.09–1.49)**	1.21(1.03–1.42)*	1.08(0.93–1.27)
Quartile 4	1.50(1.29–1.75)***	1.45(1.24–1.69)***	1.16(0.98–1.36)
*p* for trend	< 0.001	< 0.001	0.06

Model 1, no covariates were adjusted. Model 2, age, sex were adjusted. Model 3, age, sex, race, smoking, drinking, sleep, BMI, hypertension, daiabetes, metabolic index (ALT, AST, totalbilirubin, urea, creatinine, crp) or Vitamin-D or Dietary Risk Score were adjusted

Combined Group: Group 1 (Vitamin-D >  = 30 nmol/L/Dietary Risk Score =  < 4); Group 2 (Vitamin-D >  = 30 nmol/L/Dietary Risk Score > 4); Group 3 (Vitamin-D < 30 nmol/L/Dietary Risk Score =  < 4); Group 4 (Vitamin-D < 30 nmol/L/Dietary Risk Score > 4)

Vitamin-D Group: Group 1 (10–36.3 nmol/L); Group 2 (36.4–50.9 nmol/L); Group 3 (51–65.5 nmol/L); Group 4 (65.6–206 nmol/L)

Dietary Risk Score Group: Group1(1–3); Group 2 (4); Group3 (5); Group 4(6–9)

HR: hazard ratio; CI: confidence interval. * p < 0.05, ** p < 0.001, *** p < 0.0001; p < 0.05 was considered statistically significant

Similarly, we separately assessed vitamin D levels and cumulative dietary risk score. In the fully adjusted model, compared to the interval with the highest vitamin D level (Q4), the HR values for Q1-3 of MAFLD were 1.47(1.20–1.79), 1.28(1.04–1.57), 1.13(0.92–1.40). For all-cause mortality, the HR values for Q1-3 were 1.87(1.60–2.20), 1.45(1.23–1.71), 1.17(0.99–1.38) (all p for trend < 0.001). The HRs of MAFLD and mortality were 0.86(0.81–0.93) and 0.77(0.73–0.82) per one predefined unit increase in vitamin D levels in model 3. In the fully adjusted model, compared to the interval with the lowest cumulative dietary risk score (Q1), the HR values for Q2-4 of MAFLD were 0.97(0.80–1.18), 1.22(1.01–1.48), 1.38(1.14–1.68) (p for trend < 0.001), and the HR values for Q2-4 for all-cause mortality were 1.02(0.87–1.19), 1.08(0.93–1.27), 1.16(0.98–1.36), respectively (p for trend = 0.06). The HRs of MAFLD and mortality were 1.15(1.08–1.24) and 1.08(1.02–1.14) per one predefined unit increase in cumulative dietary risk score in model 3 (Table 2).

### Predictive ability comparison

**Figure. 2:**
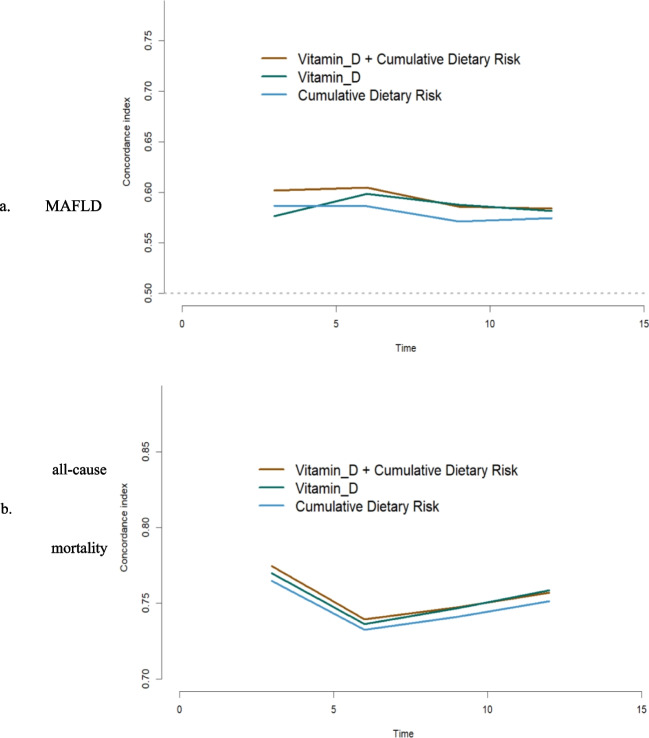
Time-dependent predictive ability of Vitamin-D, Cumulative Dietary Risk, and their combination for MAFLD (**a**), all-cause mortality (**b**). Models were adjusted for age and sex. Figure legends: Brown lines = group Vitamin-D and Cumulative Dietary Risk; Green lines = group Vitamin-D; Blue lines = group Cumulative Dietary Risk

We calculated Harrell's C-index for vitamin D, cumulative dietary risk score, and their combination over time, demonstrating that the combination of both was most strongly associated with new-onset MAFLD and all-cause mortality (Fig. 2). Additionally, we conducted subgroup analyses by gender (**Fig. S2**) and age (**Fig. S3**), revealing that the combination of vitamin D and cumulative dietary risk score exhibited the highest predictive power, except in participants aged 20–60 years with new-onset MAFLD.

### Kaplan–meier analysis

**Figure. 3:**
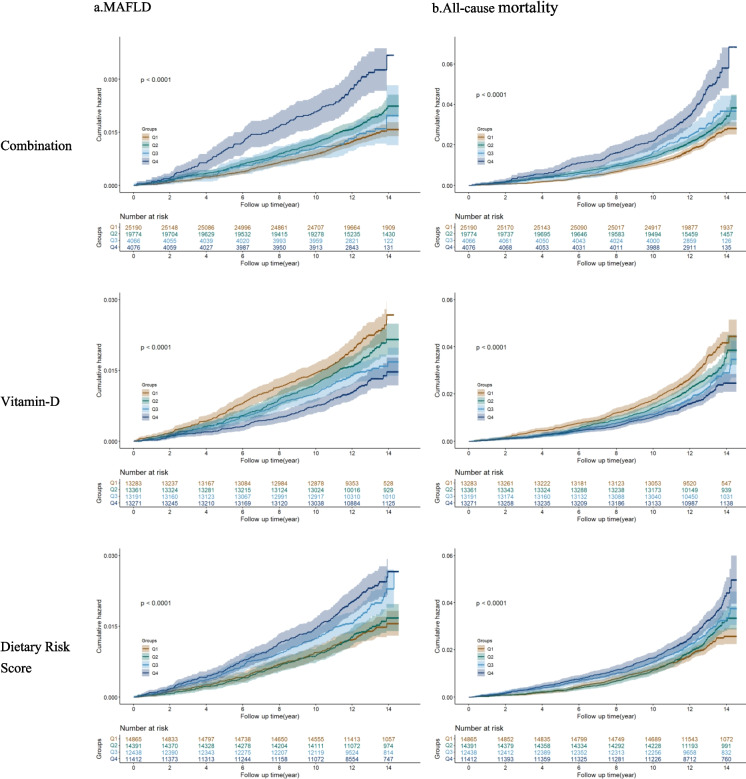
Kaplan–Meier curves of their combination, individual Vitamin-D or Dietary Risk Score. **a**) MAFLD; **b**)all-cause mortality. Models were adjusted for age and sex. Brown lines = group 1; Green lines = group 2; Light blue lines = group 3; Dark blue line = group 4

Kaplan–Meier analysis demonstrated that participants with lower vitamin D levels and higher cumulative risk scores had a significantly higher probability of developing cumulative disease, whether analyzed individually or in combination (all log-rank p < 0.001) (Fig. 3).

### Interaction and stratified analysis

We further assessed the effects of vitamin D and cumulative dietary risk scores on MAFLD and all-cause mortality by examining interactions with age, sex, ethnicity, smoking, alcohol consumption, sleep, and BMI. The results revealed no significant interactions, except for smoking, which showed an interaction with vitamin D and all-cause mortality (p for interaction = 0.047). Stratified analysis indicated that lower vitamin D levels had a more pronounced effect in current smokers, while the dietary risk score was more significant in individuals with normal weight (**Fig. S4**).

### Sensitivity analysis

**Figure. 4:**
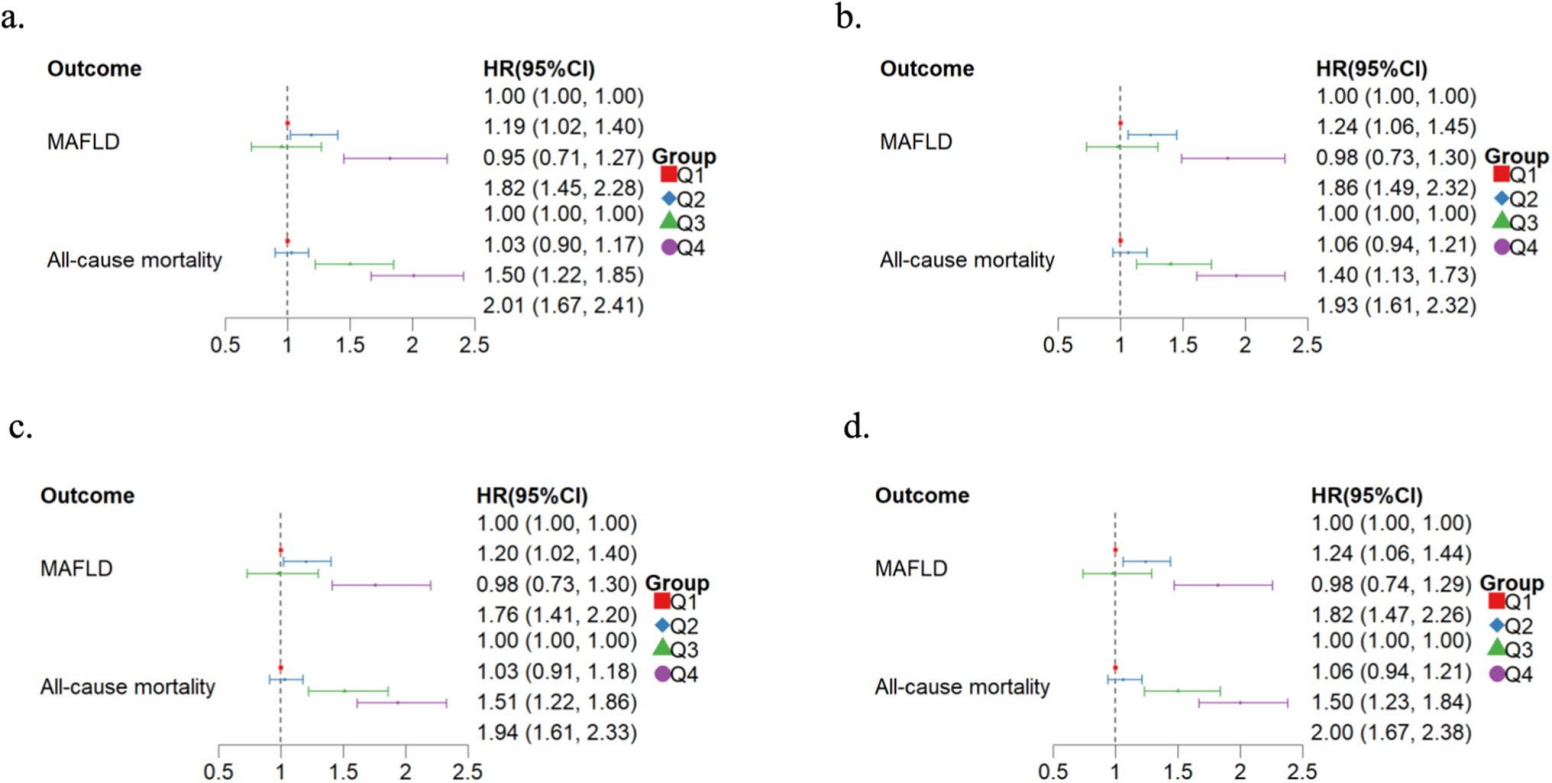
Sensitivity analysis of Combination of Vitamin-D and Dietary Risk Score for predicting MAFLD and mortality. All variables were adjusted in the Cox model, and the first quartile (Q1) served as the reference interval. **a**) Without 140 participants who had a study outcomes within 2 years. **b**) Without 1041 participants with extremely low or high Vitamin-D levels. **c**) Without 1427 participants in the acute infection phase. **d**) Median imputation instead of mean imputation

To ensure the robustness of the results, we excluded 140 participants who had experienced an outcome event in the previous two years, or 1041 with the maximum and minimum vitamin D levels. We also excluded 1427 participants in the acute infection phase and replaced mean interpolation with median interpolation. There was no substantial change in the combined effect and the outcomes (Fig. 4).

## Discussion

In this large prospective cohort study, we examined the association between combined measures of vitamin D levels and cumulative dietary risk scores with the onset of new MAFLD and all-cause mortality, and compared their predictive power over time across the general population, as well as within gender and age subgroups. Overall, the combined vitamin D and dietary risk scores demonstrated the highest predictive power for MAFLD development and all-cause death, outperforming either measure alone. Our findings emphasize the superior predictive value of multi-dimensional assessment approaches in individuals with pre-frailty or frailty. Additionally, stratified analyses revealed that fluctuations in vitamin D levels were more influential in current smokers, while the association was stronger in individuals within the normal BMI range with dietary risk scores. These results highlight the impact of lifestyle factors on health outcomes, suggesting that smoking cessation and maintaining a healthy weight can positively influence long-term health outcomes.

As individuals become more vulnerable to exogenous stress and the potential role of energy metabolism, nutrient perception, and insulin resistance, frailty phenotype significantly increases the risk of metabolic diseases and all-cause mortality [20]. The improved Fried frailty phenotype or frailty index is commonly used in frailty assessments, with the former being more frequently employed in community population studies, and the latter in inpatient populations [20]. Frailty is dynamic, which presents an opportunity for intervention, and the maintenance or reversal of fitness, pre-frailty, and frailty are influenced by multiple dimensions [21]. Previous research supports the notion that active exercise, including endurance, resistance, speed, and flexibility training, is the most effective method to improve frailty, which can improve fiber loss and fat infiltration, reduce age-related oxidative damage and chronic inflammation, increase autophagy [22]. Existing research proves that a multi-component training intervention, consisting of three sessions per week lasting 30 to 45 min for over five months, appears to be more effective in improving frailty than either individual aerobic or resistance training alone [2]. Studies have suggested that nutritional elements should be leveraged to optimize the skeletal muscle’s response to exercise metabolism and maintaining adequate vitamin D levels can support muscle protein synthesis during resistance training [22]. But research on the role of vitamin D across different types of exercise is still limited. Additionally, high-quality diet and vitamin D supplementation can also help improve or reverse frailty [7, 23]. Studies have also indicated that, in populations with widespread vitamin D deficiency, active supplementation or maintaining vitamin D at recommended levels can significantly reduce the incidence of frailty [24, 25]. A systematic analysis of 13 randomized clinical trials in individuals aged 60 and older concluded that daily vitamin D supplementation (800 IU to 1000 IU per day for 3–12 months) positively impacts muscle strength, balance, and physical function, particularly in those with baseline vitamin D deficiency or insufficiency [26]. Additionally, studies have indicated that supplementing with vitamin D (800 IU to 1000 IU per day for 3–12 months) in elderly individuals with baseline levels below 25 nmol/L, and maintaining a serum vitamin D concentration of at least 60 nmol/L, can improve muscle strength, enhance balance, and reduce the risk of falls [27]. Currently, most studies have focused on reversing frailty through interventions [28], but there is a lack of further analysis on populations that have already undergone initial interventions. The health effects of vitamin D and diet on comparably frail individuals who have already undergone exercise interventions remain unclear, and there is a need for evidence to guide the decision for a second intervention.

The two main forms of vitamin D are cholecalciferol (vitamin D3, or VD3) and ergocalciferol (vitamin D2, or VD2). In addition to its crucial role in maintaining proper calcium and phosphate levels, vitamin D is involved in cell growth, differentiation, and immune function [29]. In the liver, vitamin D can reduce fibrosis, inhibit hepatocyte apoptosis, suppress pro-inflammatory cytokines, modulate adipokine expression, and enhance bile acid transport [29]. Vitamin D deficiency is common in individuals who are relatively weak, often due to decreased outdoor activity and impaired tissue function [9]. Conversely, vitamin D can work synergistically with exercise, particularly resistance training, to enhance muscle function [22]. While most studies recommend a vitamin D concentration of 50 nmol/L [30], the conservative threshold for vitamin D deficiency is set at 30 nmol/L, due to regional and individual differences [17, 31]. Vitamin D deficiency is linked to frailty, partly due to its interaction with vitamin D receptors in skeletal muscle, which results in changes to muscle contraction and a reduction in muscle synthesis [25]. The beneficial effects of vitamin D supplementation for the elderly in improving muscle function and slowing down aging are well-established [26, 27]. In our study, we found an inverse relationship between risk and vitamin D levels, suggesting that vitamin D may have a protective effect in vulnerable populations, which provides evidence for secondary intervention.

Insufficient or unbalanced nutritional intake is a major issue faced by frail individuals, often resulting from reduced chewing function, impaired gastrointestinal digestion, and lack of proper dietary guidance [3, 4]. Due to the diversity of diets, there are various methods to assess diet quality, including the Healthy Eating Index 2015 (HEI-2015), Alternate Mediterranean Diet Score (AMED), Healthful Plant-Based Diet Index (HPDI), Alternate Healthy Eating Index (AHEI), and Dietary Approaches to Stop Hypertension (DASH) [32, 33]. In the UK, individuals following high-quality diets—such as the DASH, Mediterranean, and HEI diets—experience a reduced risk of all-cause mortality, cardiovascular disease, cancer, and cognitive decline [34, 35]. Both cross-sectional analyses and multi-ethnic cohort studies have suggested that maintaining high-quality dietary patterns, such as the HEI, AHEI, AMED, and DASH, is significantly associated with a reduced risk of MAFLD and an improvement in overall body fat, particularly in middle-aged and elderly populations [36]. A one-year Mediterranean diet intervention study conducted among elderly participants from five countries found that the Mediterranean diet was associated not only with improved frailty and cognitive function but also with beneficial changes in gut microbiota (increased short/branchchain fatty acids and reduced secondary bile acids, p-crephenol, ethanol and carbon dioxide) [37]. Poor-quality dietary patterns are positively linked to an increased risk of disease despite variations in dietary assessment methods. The cumulative dietary risk score is currently underexplored in frail populations, and the items involved are minimally influenced by geographic and disease variations, making it a more universal and actionable approach. Unlike separate dietary risk classifications, it produces a continuous variable that helps us understand non-linear correlations [16, 19]. In this study, we found a clear positive association between the cumulative dietary risk score and disease risk, as demonstrated by the J-shaped curve of RCS. However, due to the complexity and variability of dietary patterns, our study found that the predictive power of the dietary risk score alone is not superior to that of vitamin D in vulnerable people. In addition, individuals with frailty often take additional nutrients, which complicates routine dietary assessments [2].

The strength of our study is that it is the first to examine the health outcomes of combined vitamin D and dietary score in a vulnerable population with primary prevention measures, thereby addressing the question of whether the initial intervention is sufficient. Our sensitivity analysis is thorough, and the comparative tests of predictive power are adequate. However, the study does have some limitations. First, due to the lack of effective dynamic follow-up, we relied on baseline data for analysis, which may not fully capture the participants'ongoing health status [38]. Second, as the data was primarily collected through questionnaires, there is a potential for recall bias. Additionally, we did not categorize the types of exercise (aerobic training, resistance training, multi-component training) to explore the interaction between different exercise modalities and vitamin D. Furthermore, the study's sample predominantly consists of white participants, and a more comprehensive analysis would benefit from integrating data from multiple diverse cohorts. Moving forward, our goal is to conduct randomized clinical trials to further investigate the effects of vitamin D supplementation and dietary interventions on health outcomes in vulnerable populations.

## Conclusions

In populations with frailty undergoing exercise interventions, combining vitamin D supplementation with dietary risk assessment can serve as a more powerful predictor of disease risk. This approach has positive clinical implications for further lifestyle adjustments and the reduction of disease risk, providing valuable evidence to guide secondary intervention and improve health outcomes.

## Supplementary Information

Below is the link to the electronic supplementary material.

Supplementary file1 (DOCX 938 KB)

## Data Availability

The datasets generated or analyzed during the current study are not publicly available due to data protection but are available from the corresponding author on reasonable request.

## Declarations

### Guarantors

Mengchen Zou is the guarantor of this work and, as such, had full access to all the data in the study and takes responsibility for the integrity of the data and the accuracy of the data analysis.
