# Supplementary material for: Combined effects of vitamin D and cumulative dietary risk score on fatty liver and mortality in vulnerable individuals: a prospective analysis from the UK Biobank
Source: GeroScience. 2025 Jul 1;48(2):1771–85. doi: 10.1007/s11357-025-01762-y (PMC12972413; doi:10.1007/s11357-025-01762-y)
Supplement: Supplementary file 1 — Supplementary file1 (DOCX 938 KB) [file 11357_2025_1762_MOESM1_ESM.docx]

Combined Effects of Vitamin D and Cumulative Dietary Risk Score on Fatty Liver and Mortality in Vulnerable Individuals: A Prospective Analysis from the UK Biobank

***GeroScience***

Xiaoyan Wang^1^, Yongqi Liang^2^, Chenxi Jin^1^, Jingjing Liang^1^, Yining Xu^1^, Xianbo Wu^2,*^, Mengchen Zou^1,*^

Xiaoyan Wang and Yongqi Liang contributed equally to this work.

Authors’ affiliation

^1^Department of Endocrinology and Metabolism, Nanfang Hospital, Southern Medical University, Guangzhou, China

^2^Department of Occupational Health and Medicine, School of Public Health, Southern Medical University, Guangzhou, China

*Corresponding Author

^1^Mengchen Zou, Email: [zoumc163@163.com](mailto:zoumc163@163.com)

Postal Address: Department of Endocrinology and Metabolism, Nanfang Hospital, Southern Medical University, 1838 Guangzhou Road North, Guangzhou 510515, China

ORCID:0000-0003-1409-4645

^2^Xianbo Wu, Email: wuxb1010@smu.edu.cn

Postal Address: Department of Endocrinology and Metabolism, Nanfang Hospital, Southern Medical University, 1838 Guangzhou Road North, Guangzhou 510515, China

Contents：

Figures:

**Figure S1.** Flowchart of the study cohort in the UK Biobank database.

**Figure S2.** Sex‐specific time‐dependent predictive capacity of vitamin D, Cumulative Dietary Risk, and their combination for MAFLD (A) and all-cause mortality (B).

**Figure S3.** Age‐specific time‐dependent predictive capacity of vitamin D, Cumulative Dietary Risk, and their combination for MAFLD (A) and all-cause mortality (B).

**Figure S4.** Stratified analysis for relationship between Vitamin-D levels or Cumulative Dietary Risk Scores and the risk of MAFLD or all-cause mortality. p for interaction (the likelihood ratio test). Levels of significance: p <0.05. The model adjusted for all relevant covariates, with the first interval used as the reference group for subgroup analysis. The graph illustrates the hazard ratio for the fourth interval.

Tables:

**Table S1.** Definition of physical frailty in the UK Biobank.

**Table S2.** Definition of cumulative dietary risk scores in the UK Biobank.

**Table S3.** The distribution of frailty phenotype and the incidence of MAFLD and all-cause mortality in combined groups.

Participants from UK Biobank 2006-2010

N=502507

Participants without MAFLD

N=74355

Participants not diagnosed with cancer

Age above 20

with enthic smoke drink sleep data

N=53106

Diagnosed with cancer

Age under 20

enthic smoke drink sleep data missing

N=14480

diagnosed with MAFLD before recruitment

N=436

Participants with Vitamin_D data

N=67586

Vitamin_D data missing

N=6769

not diagnosed with pre-frailty or frailty before recruitment

N=319346

Participants diagnosed with pre-frailty or frailty

N=183161

not meeting recommended physical activity

N=108370

Participants meeting recommended physical activity

N=74791

Figure S1. Flowchart of the study cohort in the UK Biobank database.

|  |  | Female | Male |
| --- | --- | --- | --- |
| A. | MAFLD | 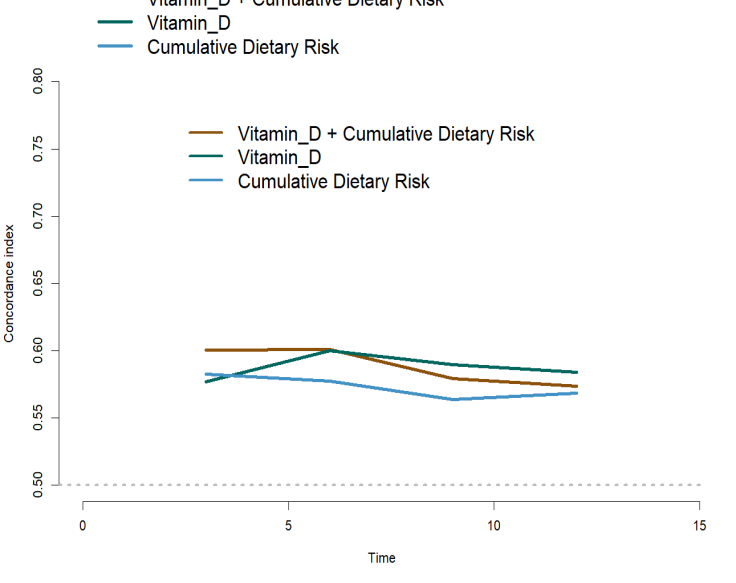 | 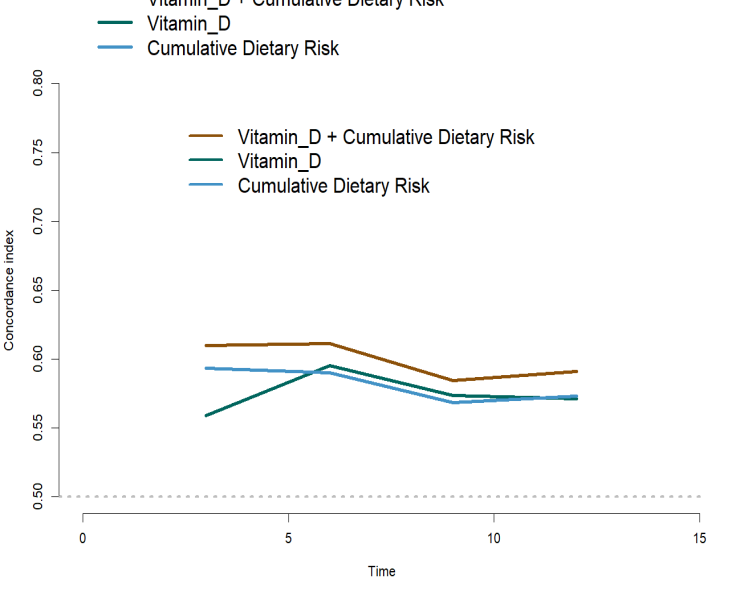 |
| B. | all-cause mortality | 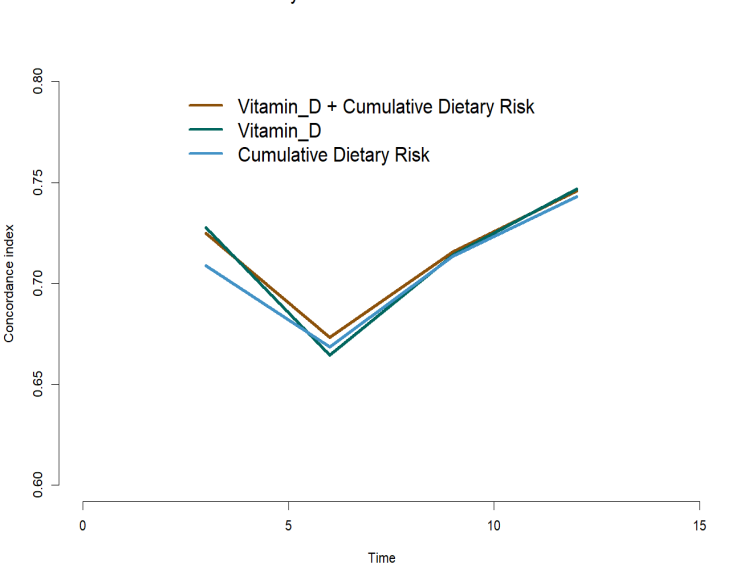 | 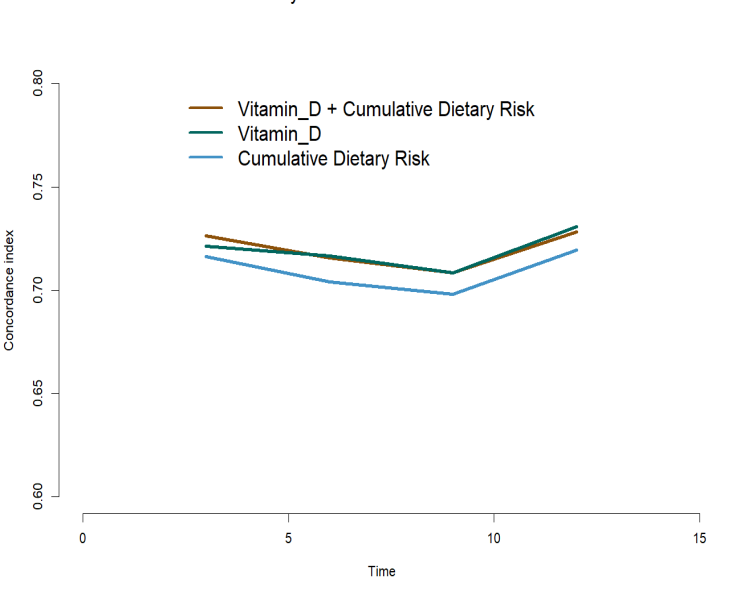 |

Figure S2: Sex‐specific time‐dependent predictive capacity of vitamin D, Cumulative Dietary Risk, and their combination for MAFLD (A) and all-cause mortality (B).

|  |  | Age:20-60 | Age:>60 |
| --- | --- | --- | --- |
| A. | MALLD | 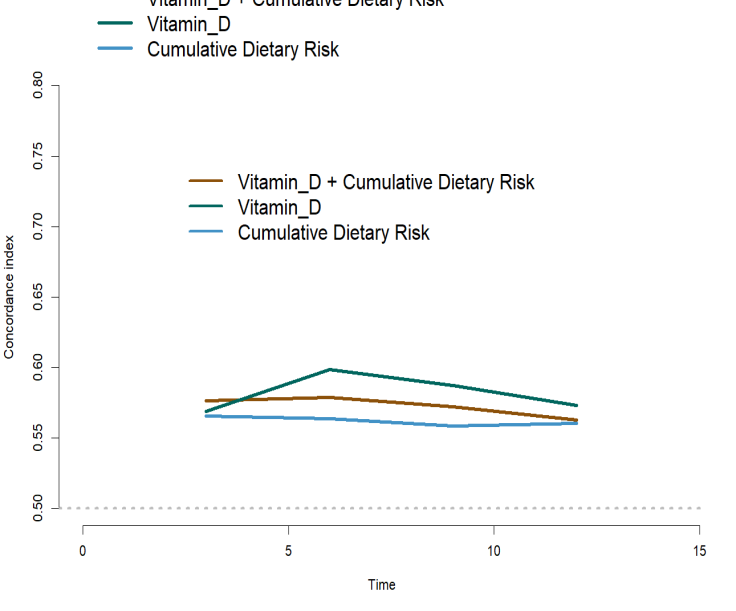 | 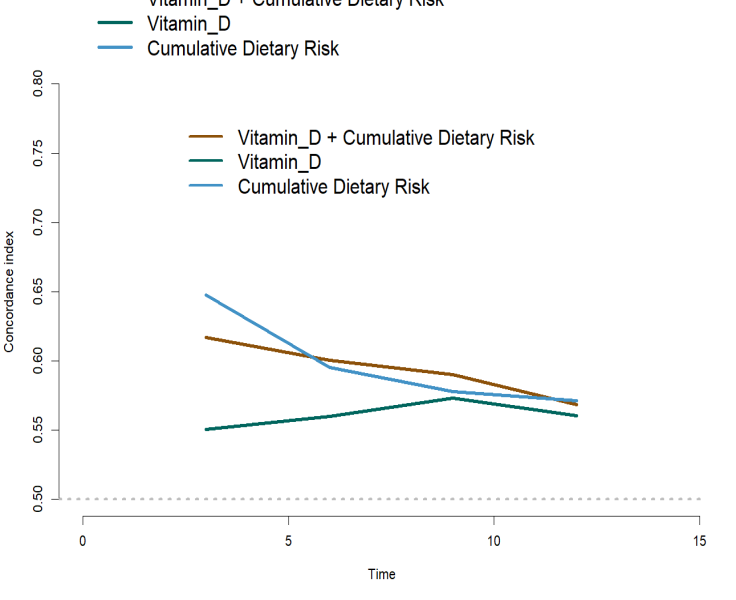 |
| B. | All-cause mortality | 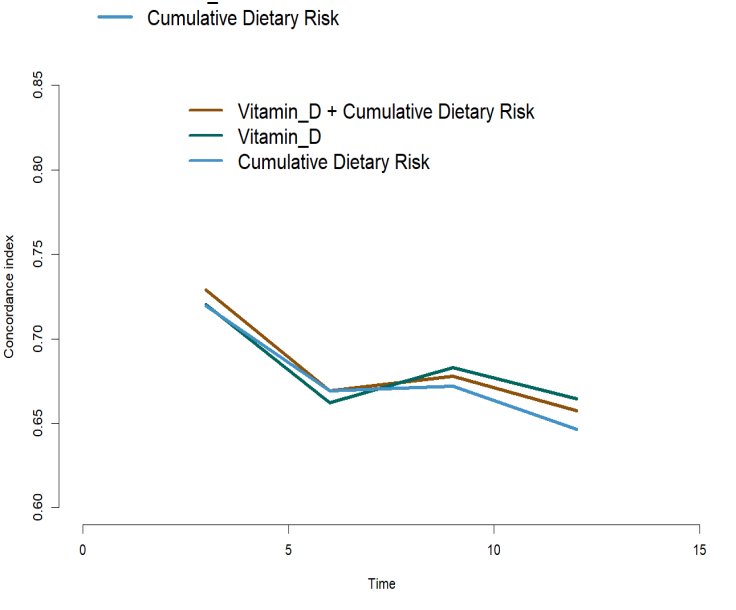 | 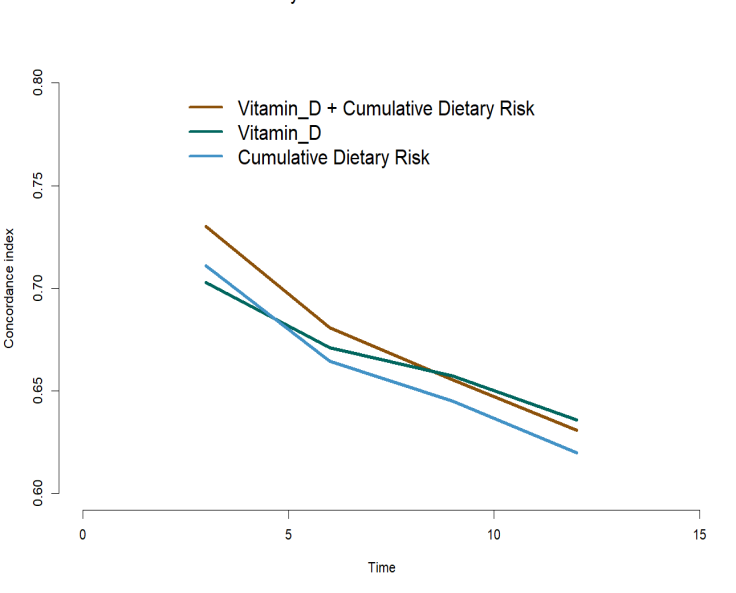 |

Figure S3: Age‐specific time‐dependent predictive capacity of Vitamin D, Cumulative Dietary Risk, and their combination for MAFLD (A) and all-cause mortality (B).

| A. | Vitamin-D | 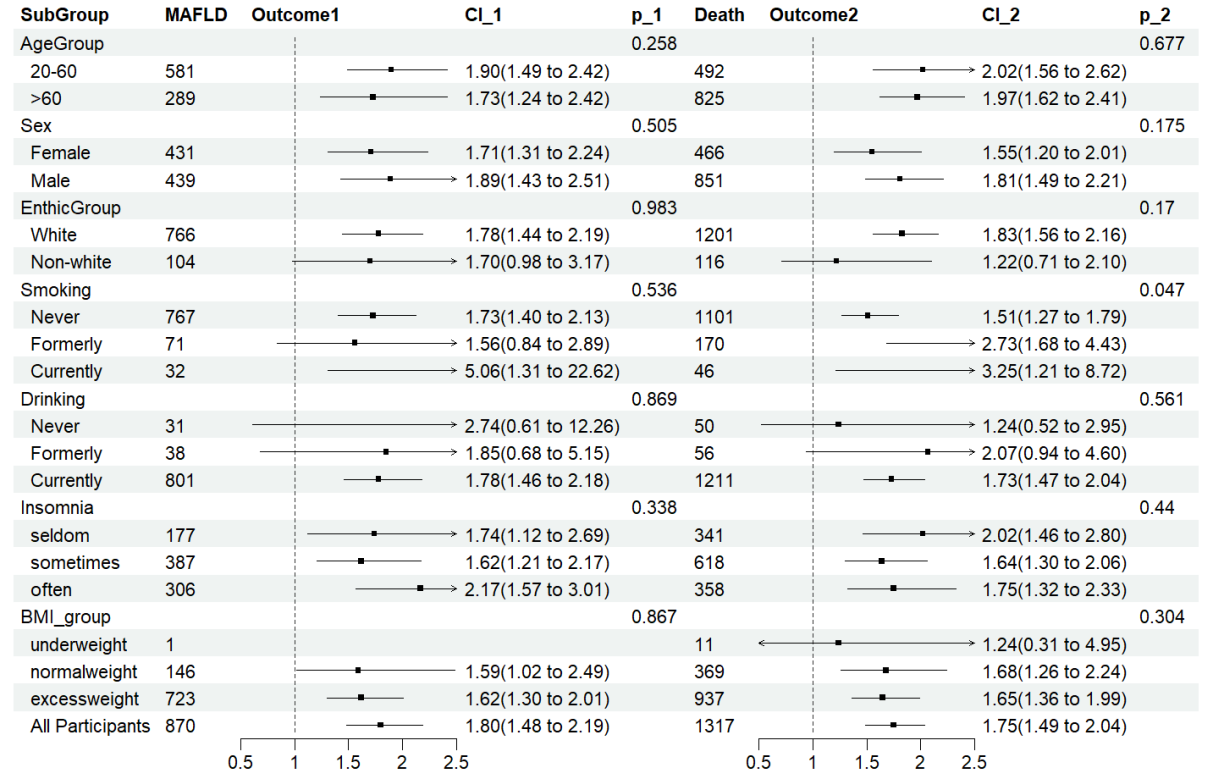 |
| --- | --- | --- |
| B. | Cumulative Dietary Risk | 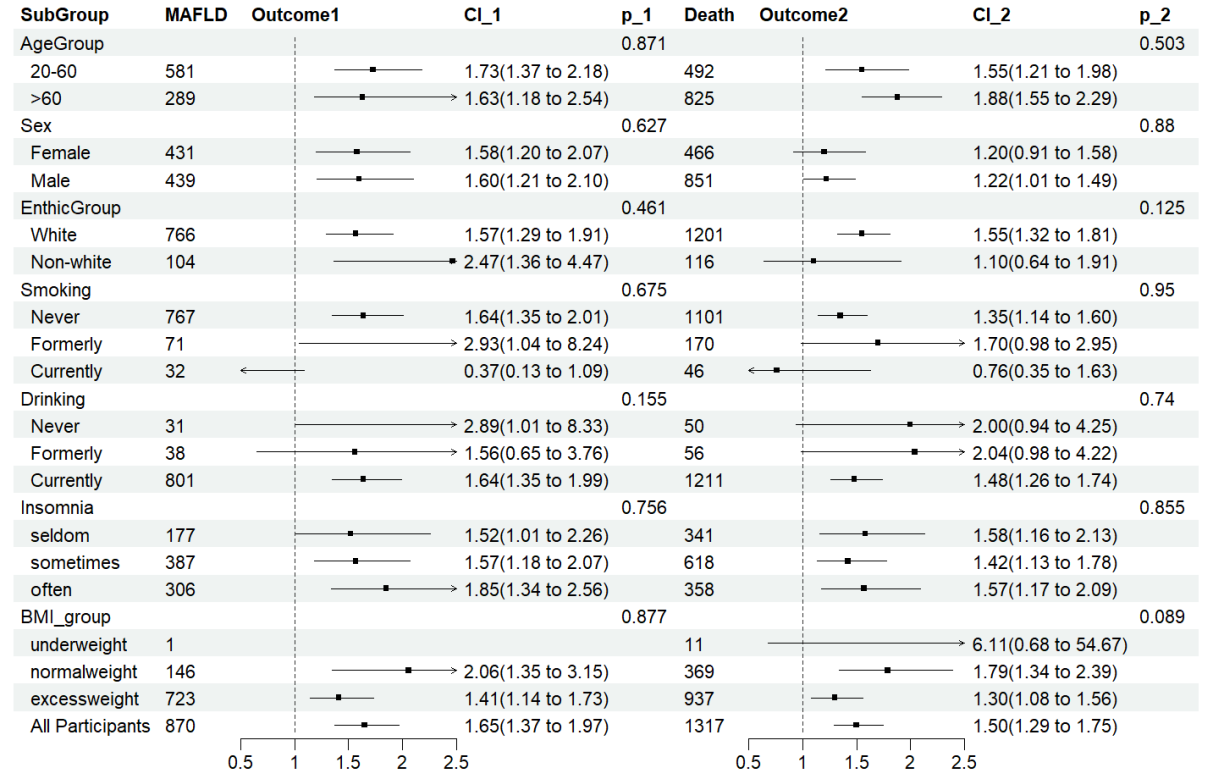 |

Figure S4: Stratified analysis for relationship between Vitamin-D levels or Cumulative Dietary Risk Scores and the risk of MAFLD or all-cause mortality. p for interaction (the likelihood ratio test). Levels of significance: p <0.05. The model adjusted for all relevant covariates, with the first interval used as the reference group for subgroup analysis. The graph illustrates the hazard ratio for the fourth interval.

Table S1. Definition of physical frailty in the UK Biobank.

| **Components** | **Self-reported question/**  **measurement in UK Biobank** | **Categories** |
| --- | --- | --- |
| Weight loss | Self-reported: “Compared with one year ago, has your weight changed?” | 1: “Yes, loss weight”;  0: Others NA: "Do not know/Prefer not to answer" |
| Exhaustion | Self-reported: “Over the past two weeks, how often have you felt tired or had little energy?” | 1: “More than half the days or nearly every day”;  0: Others; NA: "Do not know/Prefer not to answer" |
| Low physical activity | Self-reported: "How many times in the last 4 weeks did you do light DIY?" | 1: “None or light activity with a frequency of once per week or less”; 0: medium or heavy activity, or light activity more than once per week |
| Slow gait speed | Self-reported: “How would you describe your usual walking pace?” | 1: “Slow pace”;  0: Others NA: "Prefer not to answer" |
| Low grip strength | Grip strength was measured with a Jamar J00105 hydraulic hand dynamometer (Lafayette Instrument, Lafayette, IN, USA). Participants were asked to complete a grip assessment for both hands once. The maximal value of the right and left hands was used. Measured grip strength expressed in kg by sex- and BMI- adjusted cut-off points. | 1: Male:  ≤29 kg for BMI ≤24 kg/m^2^;  ≤30 kg for BMI 24.1-26 kg/m2;  ≤30 kg for BMI 26.1-28 kg/m^2^; or  ≤32 kg for BMI >28 kg/m^2^; Female:  ≤17 kg for BMI ≤23 kg/m^2^;  ≤17.3 kg for BMI 23.1-26 kg/m^2^;  ≤18 kg for BMI 26.1-29 kg/m^2^; or  ≤21 kg for BMI >29 kg/m^2^; 0: Others NA: data on BMI or grip strength is not available |

Table S2. Definition of cumulative dietary risk scores in the UK Biobank.

| Variable | Variable | Unit conversion | Binary Variables: |
| --- | --- | --- | --- |
| Fruit & vegetables (regrouped from fruit, dried fruit & Vegetable) | Fresh fruit intake (pieces/day) | Amount per serving: 1 piece 0.5 Less than one | 0 > = 5 serving/day (Ref.)  1 <5 serving/day |
|  | Dried fruit intake (pieces/day) | Amount per serving: 2 piece 0.5 Less than one |  |
|  | Cooked vegetable intake (tablespoons/day) | Amount per serving: 2 heaped tablespoons 0.5 Less than one |  |
|  | Salad / raw vegetable intake (tablespoons/day) | Amount per serving: 2 heaped tablespoons 0.5 Less than one |  |
| Total fish intake (regrouped from Both total non-oily fish and oily fish) | Oily fish intake (per week) | 0.5 Less than one 1 Once a week 3 2-4 times a week 5.5 5-6 times a week 7 Once or more daily | 0 > = 2 times a week (at least once a week of each category) (Ref.) 1 < once a week of each one |
|  | Non-oily fish intake (per week) | 0.5 Less than one 1 Once a week 3 2-4 times a week 5.5 5-6 times a week 7 Once or more daily |  |
| Processed meat intake | Processed meat intake (per week) | 0.5 Less than one 1 Once a week 3 2-4 times a week 5.5 5-6 times a week 7 Once or more daily | 0 < = Once a week (Ref.) 1 > Once a week |
| Red meat (regrouped from beef, pork and lamb) | Beef intake (per week) | 0.5 Less than one 1 Once a week 3 2-4 times a week 5.5 5-6 times a week 7 Once or more daily | 0 < = Once a week (Ref.) 1 >Once a week |
|  | Lamb/mutton intake (per week) | 0.5 Less than one 1 Once a week 3 2-4 times a week 5.5 5-6 times a week 7 Once or more daily |  |
|  | Pork intake (per week) | 0.5 Less than one 1 Once a week 3 2-4 times a week 5.5 5-6 times a week 7 Once or more daily |  |
| Milk type used | Milk type used |  | 0 Semi-skimmed/skimmed (Ref.)  1 Full cream/ another type of milk/ never rarely have milk |
| Spread type | Spread type |  | 0 Never/rarely (Ref.)  1 Another selection |
| Cereal intake * | Cereal intake (Bowls/week ) | Amount per serving: Bran/oat/muesli cereal– 1 bowl/day 0.5 Less than one | 0 >5 bowls (Ref)  1 < = 5 bowls |
| Salt added to food | Salt added to food |  | 0 Never/rarely (Ref.)  1 Another selection |
| Water intake | Water intake (Glasses/day) |  | 0 > = 6 glasses (Ref.)  1 <6 glasses |

Table S3. The distribution of frailty phenotype and the incidence of MAFLD and all-cause mortality in combined groups.

| Groups | Frailty phenotype | Events | Incidence rate(%) | Events  (MAFLD) | Incidence rate(%) | Events  (All-cause mortality) | Incidence rate(%) |
| --- | --- | --- | --- | --- | --- | --- | --- |
| Q1  (N=25190) | Prefrail | 24449 | 97.06 | 310 | 1.27 | 495 | 2.02 |
|  | Frail | 741 | 2.94 | 29 | 3.91 | 30 | 4.05 |
| Q2  (N=19774) | Prefrail | 19269 | 97.45 | 333 | 1.73 | 475 | 2.47 |
|  | Frail | 505 | 2.55 | 18 | 3.56 | 21 | 4.16 |
| Q3  (N=4066) | Prefrail | 3908 | 96.11 | 57 | 1.46 | 114 | 2.92 |
|  | Frail | 158 | 3.89 | 2 | 1.27 | 4 | 2.53 |
| Q4  (N=4076) | Prefrail | 3897 | 95.61 | 111 | 2.85 | 164 | 4.21 |
|  | Frail | 179 | 4.39 | 10 | 5.59 | 14 | 7.82 |
